# Morphological Spectrum of Vesiculobullous Skin Lesions: An Institutional Perspective

**DOI:** 10.7759/cureus.15330

**Published:** 2021-05-30

**Authors:** Javaria Ali, Sabeeh Islam, Syed Munqaad Ali, Syed Rafay Yaqeen, Anum Aslam, Qurat ul ain Khan, Umair Arshad Malik, Muhammad Irfan, Hanna Naqvi, Atif A Hashmi

**Affiliations:** 1 Pathology, Liaquat National Hospital and Medical College, Karachi, PAK; 2 Internal Medicine, Faisalabad Medical University, Faisalabad, PAK; 3 Internal Medicine, Dow University of Health Sciences, Karachi, PAK; 4 Internal Medicine, Baqai Medical University, Karachi, PAK; 5 Internal Medicine, Liaquat National Hospital and Medical College, Karachi, PAK; 6 Internal Medicine, Ashfaq Memorial Hospital, Karachi, PAK; 7 Internal Medicine, Services Institute of Medical Sciences, Lahore, PAK; 8 Internal Medicine, Aga Khan University, Karachi, PAK; 9 Statistics, Liaquat National Hospital and Medical College, Karachi, PAK

**Keywords:** vesiculobullous skin lesions, bullous pemphigoid, pemphigus vulgaris, dermatitis herpetiformis, darier’s disease, pemphigus foliaceus, epidermolysis bullosa, linear iga dermatosis, paraneoplastic pemphigus, drug reaction

## Abstract

Introduction

A vesiculobullous lesion of the skin encompasses a group of dermatological disorders with protean clinicopathological features. They usually occur as a part of the spectrum of various infectious, inflammatory, drug-induced, genetic, and autoimmune disorders. Therefore, accurate diagnosis of these lesions is essential for appropriate management and to reduce the associated morbidity and mortality. The conventional skin punch biopsy is the mainstay in the diagnosis of dermatological diseases, especially when combined with confirmatory tests, such as direct immunofluorescence (DIF). Our study evaluated the clinicopathological spectrum of vesiculobullous lesions.

Methods

We studied 150 cases of vesiculobullous lesions at the Department of Histopathology, Liaquat National Hospital and Medical College Karachi, Pakistan. Written and informed consent was taken from the patients followed by skin punch procedure in which three biopsies were obtained, which included one biopsy from the lesion and two peri-lesional biopsies. One peri-lesional biopsy was sent in cryomatrix for DIF studies, whereas the other two were sent in formalin to follow the standard tissue-processing protocol.

Results

Our results showed that most patients belonged to the geriatric age group of more than 50 years (44.7%), and 54.7% of the patients were females. Total 74.7% of the patients had generalized lesions, followed by lower limbs (9.3%) and trunk (7.3%) involvement. Most patients were diagnosed with bullous pemphigoid (31.3%), followed by pemphigus vulgaris (27.3%), dermatitis herpetiformis (15.3%), Darier’s disease (14.7%), pemphigus foliaceus (4.7%), epidermolysis bullosa (2%), linear immunoglobulin A dermatosis (2%), paraneoplastic pemphigus (0.7%), and drug reactions (0.7%). DIF studies were applied on 60 cases, out of which complement protein C3c was the most commonly deposited protein (53.3%).

Conclusion

Our study emphasized the diagnostic role of skin punch biopsy in the proper evaluation of vesiculobullous skin lesions. Histopathology is the cornerstone diagnostic tool in this regard, with DIF being a useful adjunct.

## Introduction

A vesiculobullous lesion of the skin encompasses a group of dermatological disorders with protean clinicopathological features. Vesicular lesions measure 0.5 cm or less in diameter, whereas a lesion more than 0.5 cm in diameter is classified as bulla. They usually occur as a part of the spectrum of various infectious, inflammatory, drug-induced, genetic, and autoimmune disorders [1]. Therefore, accurate diagnosis of these lesions is essential for appropriate management and to reduce the associated morbidity and mortality.

The conventional skin punch biopsy is the mainstay in the diagnosis of dermatological diseases, especially when combined with confirmatory tests, such as direct immunofluorescence (DIF) [2]. When used appropriately, DIF can also predict the relapse as well as detect lesions in remission, and, therefore, it serves as an important prognostic tool [3].

Histopathological analysis of these lesions can provide basic information, such as the mechanism of bulla formation, a type of inflammatory infiltrate (if any), level of cleavage formation, and condition of the adjacent epidermis and dermis. However, as the lesion ages, the plane of the separation and type of inflammatory infiltrate may vary and can provide a hindrance in accurate assessment and mimic variety of other non-related conditions. Our study aimed to evaluate the clinicopathological spectrum of vesiculobullous lesions.

## Materials and methods

We retrospectively studied 150 cases of vesiculobullous lesions at the Department of Histopathology, Liaquat National Hospital and Medical College Karachi, Pakistan. Clinical information obtained from the clinical referral reports included the age and sex of the patient, the type of lesion and the site, size, number, and duration of these lesions.

Written and informed consent was taken from the patients, followed by skin punch procedure in which three biopsies were obtained, which included one biopsy from the lesion and two peri-lesional biopsies. One peri-lesional biopsy was sent in cryomatrix for DIF studies, whereas the other two were sent in formalin to follow the standard tissue-processing protocol. After formalin fixation, all skin punch biopsies were bisected (perpendicular to the plane of bulla) and submitted entirely in separate cassettes, followed by vertical sectioning. The hematoxylin and eosin (H & E)-stained slides were then reviewed by a senior histopathologist. The lesions were classified according to the level of cleavage into subcorneal, intraepidermal, suprabasal, and subepidermal bulla, and the final histopathological diagnosis was made. Apart from H & E stain, periodic acid-schiff stain was performed to rule out any fungal infection.

Data analysis was performed using Statistical Package for Social Sciences (Version 26.0, IBM Inc., Armonk, NY, USA). Fisher’s exact test was used to check the association. P-values <0.05 were considered significant.

## Results

Our results showed that most patients belonged to the geriatric age group of more than 50 years (44.7%), and 54.7% of the patients were females. Total 74.7% of the patients had generalized lesions, followed by lower limbs (9.3%) and trunk (7.3%). Most patients were diagnosed with bullous pemphigoid (31.3%), followed by pemphigus vulgaris (27.3%), dermatitis herpetiformis (15.3%), Darier’s disease (14.7%), pemphigus foliaceus (4.7%), epidermolysis bullosa (2%), linear immunoglobulin A (IgA) dermatosis (2%), paraneoplastic pemphigus (0.7%), and drug reactions (0.7%). DIF studies were applied in 60 cases, out of which complement C3c protein was the most commonly deposited protein (53.3%) (Table 1).

**Table 1:** Clinicopathological characteristics of population under study SD, standard deviation; Ig, immunoglobulin; C, complement.

Clinicopathological characteristics	Values
Age (years), mean±SD	48.87±20.67
Age group	
<18 years, n (%)	11 (7.3)
18-35 years, n (%)	26 (17.3)
36-50 years, n (%)	46 (30.7)
>50 years, n (%)	67 (44.7)
Gender	
Male, n (%)	68 (45.3)
Female, n (%)	82 (54.7)
Site	
Generalized, n (%)	112 (74.7)
Upper limbs, n (%)	8 (5.3)
Lower limbs, n (%)	14 (9.3)
Trunk, n (%)	11 (7.3)
Abdomen, n (%)	2 (1.3)
Back, n (%)	1 (0.7)
Face/neck, n (%)	2 (1.3)
Level of bulla	
Subcorneal, n (%)	6 (4)
Intraepidermal, n (%)	2 (1.3)
Suprabasal, n (%)	44 (29.3)
Subepidermal, n (%)	98 (65.3)
Direct immunofluorescence	
Performed, n (%)	60 (40)
Not performed, n (%)	90 (60)
IgA (n=60)	
Positive, n (%)	4 (6.7)
Negative, n (%)	56 (93.3)
IgG (n=60)	
Positive, n (%)	24 (40)
Negative, n (%)	36 (60)
IgM (n=60)	
Positive, n (%)	8 (13.3)
Negative, n (%)	52 (86.7)
C3c (n=60)	
Positive, n (%)	32 (53.3)
Negative, n (%)	28 (46.7)
C1q (n=60)	
Positive, n (%)	8 (13.3)
Negative, n (%)	52 (86.7)
Diagnosis	
Pemphigus vulgaris, n (%)	41 (27.3)
Paraneoplastic pemphigus, n (%)	1 (0.7)
Epidermolysis bullosa, n (%)	3 (2)
Drug reaction, n (%)	1 (0.7)
Erythema multiforme, n (%)	2 (1.3)
Bullous pemphigoid, n (%)	47 (31.3)
Dermatitis herpetiformis, n (%)	23 (15.3)
Linear IgA dermatosis, n (%)	3 (2)
Pemphigus foliaceus, n (%)	7 (4.7)
Darier’s disease, n (%)	22 (14.7)

Table 2 shows the association of vesiculobullous lesions with age, gender, and site. The most common entities seen in patients younger than 18 years were bullous pemphigoid, dermatitis herpetiformis, epidermolysis bullosa, and linear immunoglobulin A (IgA) dermatosis. For age groups 18-35 and 36-50 years, pemphigus vulgaris was the most common disorder, whereas bullous pemphigoid was the most common entity for ages 50 years and older, followed by Darier’s disease. The gender distribution for pemphigus vulgaris was almost equal and it had a generalized distribution (65.9%) of lesions all over the body, followed by trunk (12.2%). Most patients with bullous pemphigoid and Darier’s disease were females (63.8 and 63.6%, respectively), having a generalized distribution of lesions (85.1% and 68.2%, respectively). The remaining entities showed male gender preferences (statistically insignificant) with majority having generalized lesions; however, lesions of erythema multiforme were commonly observed in the upper limbs and trunk, and the difference was not statistically significant (Table 2).

**Table 2:** Association of clinicopathological characteristics with final diagnosis *Fisher’s exact test was applied, **significant at <0.05. IgA, immunoglobulin A.

Clinicopathological characteristics	Values										P-value
	Diagnosis										
	Pemphigus vulgaris	Paraneoplastic pemphigus	Epidermolysis bullosa	Drug reaction	Erythema multiforme	Bullous pemphigoid	Dermatitis herpetiformis	Linear IgA dermatosis	Pemphigus foliaceus	Darier’s disease	
Age group*											
<18 years, n (%)	0 (0)	0 (0)	2 (66.7)	0 (0)	0 (0)	3 (6.4)	3 (13)	2 (66.7)	0 (0)	1 (4.5)	<0.0001**
18-35 years, n (%)	8 (19.5)	1 (100)	1 (33.3)	0 (0)	2 (100)	4 (8.5)	4 (17.4)	1 (33.3)	2 (28.6)	3 (13.6)	
36-50 years, n (%)	22 (53.7)	0 (0)	0 (0)	0 (0)	0 (0)	7 (14.9)	8 (34.8)	0 (0)	4 (57.1)	5 (22.7)	
>50 years, n (%)	11 (26.8)	0 (0)	0 (0)	1 (100)	0 (0)	33 (70.2)	8 (34.8)	0 (0)	1 (14.3)	13 (59.1)	
Gender*											
Male, n (%)	21 (51.2)	1 (100)	2 (66.7)	1 (100)	2 (100)	17 (36.2)	12 (52.2)	2 (66.7)	2 (28.6)	8 (36.4)	0.339
Female, n (%)	20 (48.8)	0 (0)	1 (33.3)	0 (0)	0 (0)	30 (63.8)	11 (47.8)	1 (33.3)	5 (71.4)	14 (63.6)	
Site*											
Generalized, n (%)	27 (65.9)	1 (100)	3 (100)	1 (100)	0 (0)	40 (85.1)	17 (73.9)	3 (100)	5 (71.4)	15 (68.2)	0.926
Upper limbs, n (%)	3 (7.3)	0 (0)	0 (0)	0 (0)	1 (50)	3 (6.4)	0 (0)	0 (0)	0 (0)	1 (4.5)	
Lower limbs, n (%)	2 (4.9)	0 (0)	0 (0)	0 (0)	0 (0)	3 (6.4)	4 (17.4)	0 (0)	1 (14.3)	4 (18.2)	
Trunk, n (%)	5 (12.2)	0 (0)	0 (0)	0 (0)	1 (50)	1 (2.1)	1 (4.3)	0 (0)	1 (14.3)	2 (9.1)	
Abdomen, n (%)	2 (4.9)	0 (0)	0 (0)	0 (0)	0 (0)	0 (0)	0 (0)	0 (0)	0 (0)	0 (0)	
Back, n (%)	1 (2.4)	0 (0)	0 (0)	0 (0)	0 (0)	0 (0)	0 (0)	0 (0)	0 (0)	0 (0)	
Face/neck, n (%)	1 (2.4)	0 (0)	0 (0)	0 (0)	0 (0)	0 (0)	1 (4.3)	0 (0)	0 (0)	0 (0)	

Table 3 shows the association of level of bulla with the diagnosis. A significant association of level of bulla was noted with the diagnosis, as all cases of pemphigus vulgaris showed suprabasal bullous formation, whereas all cases of bullous pemphigoid exhibited subepidermal bullous formation.

**Table 3:** Association of level of the bulla with the final diagnosis *Fisher’s exact test was applied, **significant at <0.05. IgA, immunoglobulin A.

Level of bulla*	Values										P-value
	Diagnosis										
	Pemphigus vulgaris	Paraneoplastic pemphigus	Epidermolysis bullosa	Drug reaction	Erythema multiforme	Bullous pemphigoid	Dermatitis herpetiformis	Linear IgA	Pemphigus foliaceus	Darier’s disease	
Subcorneal, n (%)	0 (0)	0 (0)	0 (0)	0 (0)	0 (0)	0 (0)	0 (0)	0 (0)	5 (71.4)	1 (4.5)	<0.0001**
Intraepidermal, n (%)	0 (0)	0 (0)	0 (0)	0 (0)	0 (0)	0 (0)	0 (0)	0 (0)	2 (28.6)	0 (0)	
Suprabasal, n (%)	41 (100)	1 (100)	0 (0)	0 (0)	0 (0)	0 (0)	0 (0)	0 (0)	0 (0)	2 (9.1)	
Subepidermal, n (%)	0 (0)	0 (0)	3 (100)	1 (100)	2 (100)	47 (100)	23 (100)	3 (100)	0 (0)	19 (86.4)	

Table 4 shows the association of diagnosis with DIF studies. Complement protein C3c and IgG were the most frequently positive complement protein and antibody deposited; however, no significant association of DIF studies was noted with the diagnosis.

**Table 4:** Association of direct immunofluorescence studies with the final diagnosis *Fisher’s exact test was applied. Ig, immunoglobulin; C, complement.

Direct immunofluorescence*	Values										P-value
	Diagnosis										
	Pemphigus vulgaris	Paraneoplastic pemphigus	Epidermolysis bullosa	Drug reaction	Erythema multiforme	Bullous pemphigoid	Dermatitis herpetiformis	Linear IgA dermatosis	Pemphigus foliaceus	Darier’s disease	
IgA											
Positive, n (%)	2 (10)	0 (0)	0 (0)	0 (0)	0 (0)	0 (0)	0 (0)	1 (50)	0 (0)	1 (9.1)	0.298
Negative, n (%)	18 (90)	1 (100)	1 (100)	1 (100)	0 (0)	18 (100)	6 (100)	1 (50)	0 (0)	10 (90.9)	
IgG											
Positive, n (%)	10 (50)	1 (100)	0 (0)	1 (100)	0 (0)	8 (44.4)	1 (16.7)	0 (0)	0 (0)	3 (27.3)	0.351
Negative, n (%)	10 (50)	0 (0)	1 (100)	0 (0)	0 (0)	10 (55.6)	5 (83.3)	2 (100)	0 (0)	8 (72.7)	
IgM											
Positive, n (%)	3 (15)	0 (0)	0 (0)	0 (0)	0 (0)	1 (5.6)	0 (0)	1 (50)	0 (0)	3 (27.3)	0.403
Negative, n (%)	17 (85)	1 (00)	1 (100)	1 (100)	0 (0)	17 (94.4)	6 (100)	1 (50)	0 (0)	8 (72.7)	
C3c											
Positive, n (%)	12 (60)	1 (100)	1 (100)	1 (100)	0 (0)	10 (55.6)	2 (33.3)	0 (0)	0 (0)	5 (45.5)	0.569
Negative, n (%)	8 (40)	0 (0)	0 (0)	0 (0)	0 (0)	8 (44.4)	4 (66.7)	2 (100)	0 (0)	6 (54.5)	
C1q											
Positive, n (%)	1 (5)	0 (0)	0 (0)	0 (0)	0 (0)	3 (16.7)	1 (16.7)	0 (0)	0 (0)	3 (27.3)	0.627
Negative, n (%)	19 (95)	1 (100)	1 (100)	1 (100)	0 (0)	15 (83.3)	5 (83.3)	2 (100)	0 (0)	8 (72.7)	

## Discussion

In this study, bullous pemphigoid, followed by pemphigus vulgaris, was found to be the most frequent cause of vesiculobullous skin lesions in our study population. Pavani et al. studied 42 cases of vesiculobullous skin lesions. Similar to our findings, they found bullous pemphigoid to be the most common vesiculobullous skin lesion (38.1%), followed by pemphigus vulgaris. They concluded that skin punch biopsy is a reliable technique to diagnose vesiculobullous skin lesions, with DIF as an adjunctive tool [4]. Alternatively, in another study, involving 68 cases of vesiculobullous skin lesions, pemphigus vulgaris was the most common histopathological diagnosis. They claimed that characteristic histological features were present in 38.2% cases, while 17.7% cases showed nonspecific changes. Moreover, DIF was negative in 34.9% cases. Therefore, they concluded that clinical, histological, and DIF methods, in combination, are helpful in establishing a final diagnosis of a vesiculobullous skin lesion, while in isolation these methods are not diagnostic in every case [5]. In another study, apart from DIF, indirect immunofluorescence and Tzank smears were combined as adjunctive tools. A total of 34 cases were included, and pemphigus vulgaris was the most frequent diagnosis, followed by bullous pemphigoid and linear IgA dermatosis [6]. Basu studied 34 cases of intraepidermal vesiculobullous skin lesions and emphasized the role of DIF as a useful adjunctive diagnostic tool, while they found pemphigus vulgaris to be the most common entity causing intraepidermal bullous skin lesions [7].

Vesiculobullous skin lesions show geographic and ethnic variations. We found pemphigus vulgaris to be the second most frequent cause of vesiculobullous skin lesions in our study population. Pemphigus vulgaris has a higher incidence in Indian, Southeast European, and Middle Eastern populations [8]. Bullous pemphigoid is the most prevalent blistering skin disease that is autoimmune in nature, characterized by auto antibodies against hemidesmosomal antigens. Bullous pemphigoid is more common in elderly population, as noted in our study. The incidence of this disease is 7.63 per 100,000 person-years [9]. Very few studies were conducted in Pakistan to determine the relative frequencies of different vesiculobullous skin lesions.

The limitations of our study include single-institution data. Moreover, DIF was performed in less than half of the cases. Therefore, more large-scale studies are recommended to determine the relative frequencies of vesiculobullous skin lesions in our population.

## Conclusions

In this study, we determined the relative frequencies of various vesiculobullous skin lesions and found bullous pemphigoid to be the most common entity, followed by pemphigus vulgaris. Our study also underscored the diagnostic value of skin punch biopsy and histopathology, as histological findings were diagnostic in most of the cases in our study. The level of bulla formation is the most important histological parameter suggesting a possible diagnosis. The DIF is a useful adjunctive tool in difficult cases, but it is not necessary in every case.
